# Supplementary material for: Long-Term Corrosion of Eutectic Gallium, Indium, and Tin (EGaInSn) Interfacing with Diamond
Source: Materials (Basel). 2024 Jun 2;17(11):2683. doi: 10.3390/ma17112683 (PMC11174033; doi:10.3390/ma17112683)
Supplement: Supplementary file 1 [file materials-17-02683-s001.zip › materials-3009814-supplementary.pdf]

materials

MDPI

*Supplementary*

# Long-Term Corrosion of Eutectic Gallium, Indium, and Tin (EGaInSn) Interfacing with Diamond

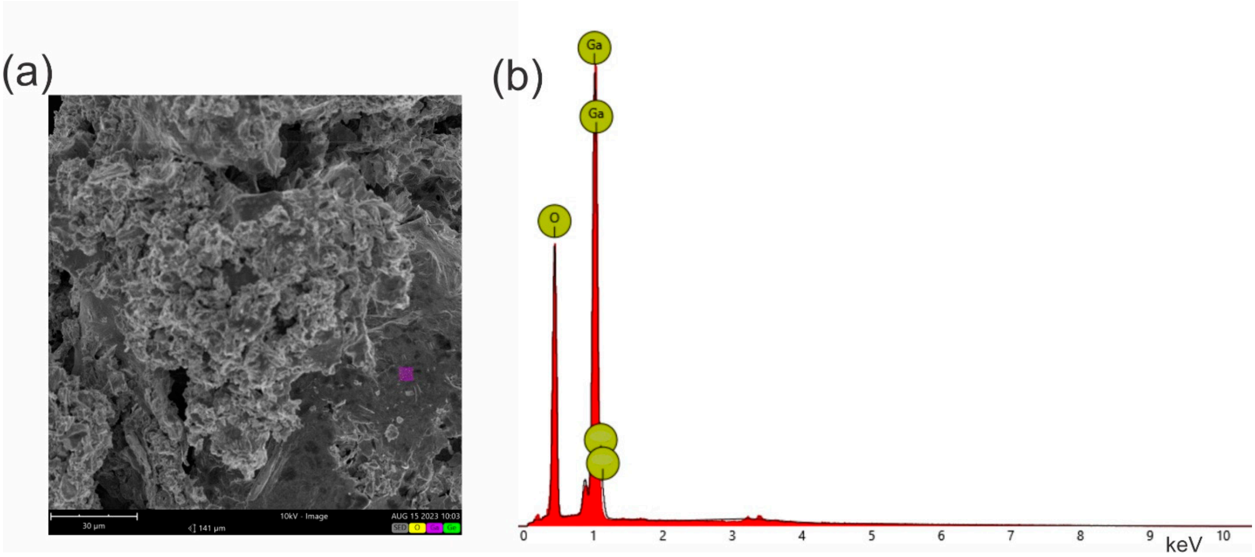


**Figure S1.** (**a**) SEM micrograph and (**b**) EDS spectrum of liquid metal after aging for 4 years on diamond coated Ti sample.

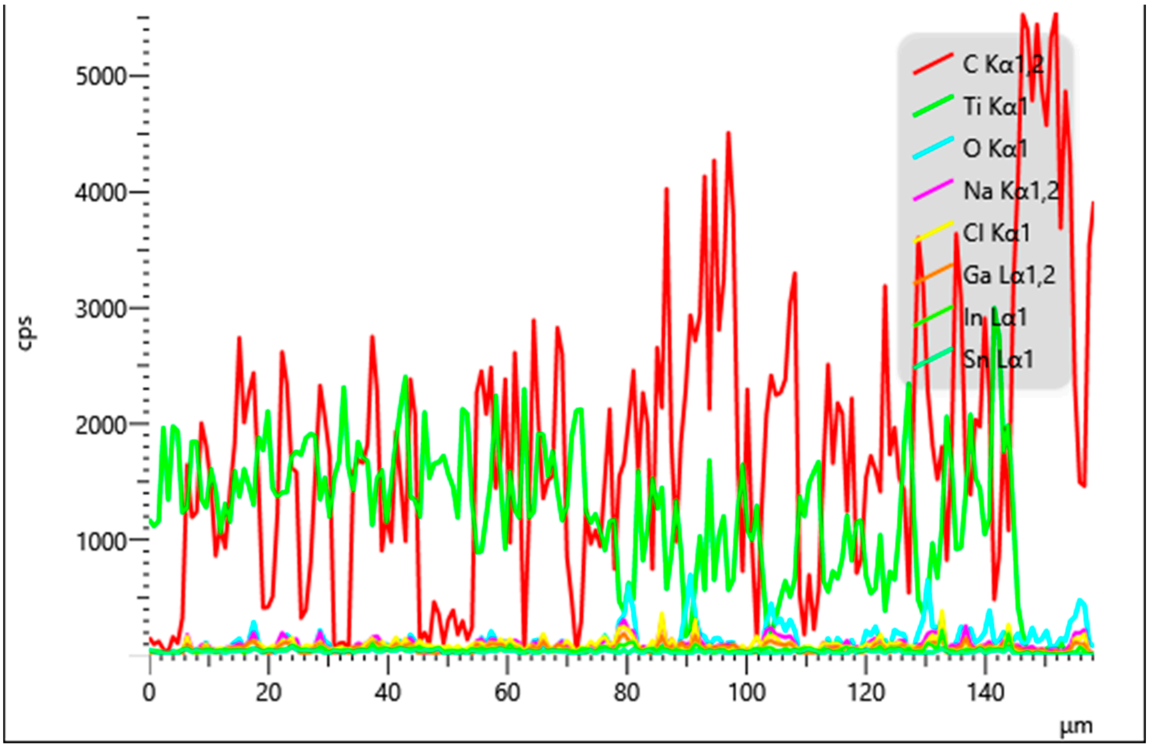


**Figure S2.** EDS line measurement of the structured diamond coated titanium alloy after exposition to liquid metal for 4 years (and cleaning).

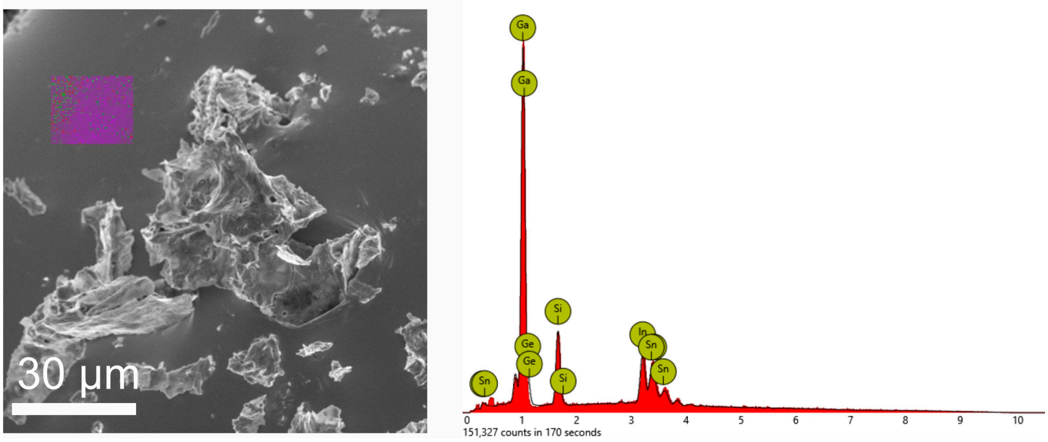


**Figure S3.** SEM image and EDS spectrum of EGaInSn on the Si sample. The purple square denotes the location the EDS spectrum was measured.

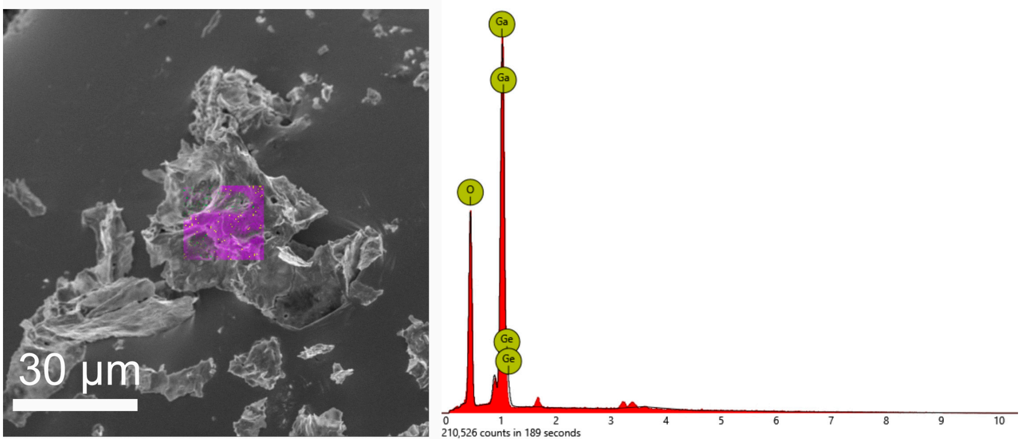


**Figure S4.** SEM image and EDS spectrum of EGaInSn on the Si sample. The purple square denotes the location the EDS spectrum was measured.
